# Applicability of the Moyers' Probability Tables in Adolescents with Different Facial Biotypes

**DOI:** 10.2174/1874210601711010213

**Published:** 2017-04-26

**Authors:** Jorge J. Pavani Carrillo, Maria C. Rubial, Cristina Albornoz, Silvina Villalba, Patricia Damiani, Marta Rugani de Cravero

**Affiliations:** Faculty of Dentistry, National University of Córdoba, Cordoba, Argentina

**Keywords:** Mesiodistal size, Moyer's probability tables, Mixed dentition analysis, Facial biotypes, Dolichofacial

## Abstract

**Introduction:**

The Moyers’ probability tables are used in mixed dentition analysis to estimate the extent of space required for the alignment of canines and premolars, by correlating the mesiodistal size of lower incisors with the size of permanent canines and premolars.

**Objective:**

This study intended to evaluate the applicability of the Moyer's probability tables for predicting the mesiodistal space needed for the correct location of premolars and permanent canines non-erupted, in adolescents of the city of Cordoba, Argentina, who show different facial biotypes.

**Materials and Methods:**

Models and tele-radiographies of 478 adolescents of both genders from 10 to 15 years of age were analyzed. The tele-radiographies were measured manually in order to determine the facial biotype. The models were scanned with a gauged scanner (HP 3670) and measured by using Image Pro Plus 4.5 software.

**Results:**

According to this study, the comparison between the Moyer´s probability table, and the table created at the National University of Córdoba (UNC) (at 95%, 75%, and 50%) shows that, in both tables, a higher value of mesiodistal width of lower incisors corresponds to a bigger difference in the space needed for permanent canines and premolars; being the need for space for permanents canines and premolars bigger in the UNC´s table. On the other hand, when contrasting the values of mesiodistal space for permanent canines and premolars associated with each facial biotype, the discrepancies between groups were not statistically significant (*P* >0.05). However, we found differences in the size of the space required according to the mesiodistal width range of the lower incisors for each biotype:

a) The comparison of lower-range values, with a mesialdistal width of lower incisors less than 22 mm, the space required for permanent canines and premolars resulted smaller in patients with dolichofacial biotype than in patients with mesofacial and braquifacial biotypes. The latter biotypes have meager differences between them.

b) The comparison of mid-range values, with a mesialdistal width of lower incisors from 22 to 25 millimeters, shows that the values of required alignment space are similar in the three facial biotypes.

c) Finally, the comparison of upper range values, with a mesialdistal width of lower incisors greater than 25 millimeters, indicates that the space required for dolichofacial biotypes tends to be higher than in mesofacial and brachyfacial biotypes.

**Conclusion:**

The Moyer´s probability tables should be created to meet the needs of the population under study, with no consideration of patients’ facial biotypes.

## INTRODUCTION

The tables used for predicting the space needed for permanent canines and premolars were published by R. Moyers in 1958 [1]. These are used in mixed dentition analysis to estimate the mesiodistal space necessary for the correct location of non-erupted upper and lower permanent canines and premolars. This necessary space is obtained by correlating the mesiodistal size of lower permanent incisors with the size of permanent canines and premolars. The diagnosis is one of the main objectives of health science. Mixed-dentition analysis is a part of orthodontic diagnosis, and it is extremely important in determining what treatment plan should be applied. This may include maintenance, recovery and supervision of space; or serial extractions [2]. Basically, three methods for the prediction of mesiodistal size of permanent canines and premolars have been published and used in different analyses of mixed dentition:

- Size measurement of non-erupted teeth, by using X-rays with or without prediction methods [3-5].
- Calculations based on tables and prediction equations that take into consideration the preexisting correlation with other erupted permanent teeth [6-9].
- A combination of X-ray measurements and prediction tables [10-13].

From the methods outlined above, the most widely used are those structured around calculations based on tables and prediction equations, which take into consideration the preexisting correlation with other erupted permanent teeth. Among these, Moyers’ analysis is often preferred. Our research was therefore focused on the prediction method used by Moyers, which uses correlation probability tables between lower incisor sizes and combined sizes of permanent canines and premolars, to predict the amount of space required for non-erupted teeth. Moyers’ tables were published for the first time in 1958 [1]. In said publication, he discussed the mesiodistal size of permanent teeth by using a table created for the thesis “Tooth Size and Symmetry in Human Dentition” by Griewe, P.W. at the University of the State of Iowa in 1949. In 1976, Moyers modified said table by also taking into account the contributions and tables provided by the Center for Human Growth and Development at the University of Michigan [14].

It is important to remark the actual words published by Moyers in his last book [15], “The tables used here are based on size variations and relationships in teeth of white Americans and they may or may not be valid for other ethnic groups.” The purpose of this research arose from Moyers’ own words, and from the absence of any similar research on this topic in Argentina; and consisted in verifying the viability of Moyers’ probability tables regarding the size of non-erupted permanent-canines and premolars, and the correlation between the size of these teeth and the facial biotype, in adolescents of both genders from the city of Córdoba, Argentina.

## MATERIALS AND METHODS

Models and teleradiographies of 478 adolescents of the city of Córdoba, Argentina; both male and female between 10 and 15 years of age (age average of 12.5 years) who concurred to the Chair Integral of Children and Adolescents “A”, Faculty of Dentistry, at the National University of Córdoba were analyzed. An informed consent form was drafted and signed by both parents of each patient. The consent included detailed information about the work to be performed and requested authorization to take the impressions and teleradiographs needed for the research.

1. The sample inclusion criteria were as follows:
  - Both parents born in Argentina
  - Permanent erupted dentition in both dental arches, except for the second and third molars.
2. The exclusion criteria were the following:
  - Discrepancy of size dental intermaxillary.
  - Agenesis.
  - Cavities, restorations, loss or fractures interproximal.
  - Congenital anomalies.
  - Previous orthodontic treatment.

Impressions were taken with alginate and cast with white stone plaster. The resulting cast models were digitized with a gauged HP 3670 scanner and the mesiodistal diameter of the teeth were measured with Image Pro Plus 4.5 software, in the Oral Biology Department of the Faculty of Dentistry of the National University of Córdoba (UNC). On the cast, the width of mesiodistal lower incisors was measured, along with the size of permanent canines and lower and upper premolars. Then the correlation was observed between the size of the lower incisors and the size of the permanents canines, and upper and lower premolars that had erupted in each of the patients.

The tele-radiographies were taken with Orthopantomograph Planmeca Pro plus, and measured manually by using the Börk-Jarabak analysis with the following percentages: for dolichofacials, 54% to 58%; mesofacial, 59% 63% and brachyfacial, 64% to 68%, in order to determine the facial biotype. These measurements were made by experienced staff at the Chair Integral of Children and Adolescents “A”, Area of Orthodontics, Faculty of Dentistry, UNC. This percentage was obtained with the aim of determining whether different facial biotypes had any significant influence on the space required for each tooth.

The values obtained in this research were later compared with Moyer’s probability tables at 50%, 75%, and 95% percentiles, with the purpose of determining the viability of using Moyers’ tables with the population of Cordoba, Argentina, and the need for the ideation of new tables that are better suited to each facial biotype. The following statistical analyses with a fixed 95% confidence level, since the value of *P* is fixed at 0.05 were carried out:

- Descriptive statistics with central tendency values on the size of each dental element in the sample.
- The Pearson’s Test was used for correlativity analysis between the following variables: size of lower permanent incisive and size of upper and lower permanents canines and premolars.
- Linear regression analysis was used to determine the probability function in the prediction table.
- Descriptive statistics with central tendency values were used for each facial typology.
- One-way ANOVA was applied to the contrast between biotypes.
- Student T-test was used to contrast values recorded by Moyers with those obtained in this research for the three percentiles (50%, 75%, and 95%) in the particular ranges of lower incisive sizes. The sex variable was also considered.

## RESULTS

1. The comparison of the two probability tables used in this study, (Moyers and UNC) at 95%, 75%, and 50% (Tables **1**, **2**, **3** and **4**) shows that, in both tables, a higher value of mesiodistal width of lower incisors corresponds to a bigger difference in the space needed for permanent canines and premolars; being the need for space for permanents canines and premolars bigger in the UNC´s table. These differences are most evident in the upper arch.
2. Specific Moyers’ probability tables, for males and females, differed significantly from those obtained in this research. The space required was greater in the UNC tables, for both genders (Tables **1**, **2**, **3** and **4**).
3. We found differences in the size of the space required according to the mesiodistal width range of the lower incisors for each biotype (Tables **5**, **6**):
  - The comparison of lower-range values, with a mesialdistal width of lower incisors less than 22 mm, the space required for permanent canines and premolars resulted smaller in patients with dolichofacial biotype than in patients with mesofacial and braquifacial biotypes. The latter biotypes have meager differences between them.
  - The comparison of mid-range values, with a mesialdistal width of lower incisors from 22 to 25 millimeters, shows that the values of required alignment space are similar in the three facial biotypes.
  - Finally, the comparison of upper range values, with a mesial-distal width of lower incisors greater than 25 millimeters, indicates that the space required for dolichofacial biotypes tends to be higher than in mesofacial and brachyfacial biotypes.

### Comparative Probability Tables:

Comparison of values estimated by Moyers and by UNC Department for female patients. Space value (upper permanent canines and premolars) according to the sum of the mesiodistal diameters of lower permanent incisors [mm], (95%, 75%, and 50%) (Table **1**).

### Comparative Probability Tables:

Comparison of values estimated by Moyers and by UNC Department for female patients. Space value (lower permanent canines and premolars) according to the sum of the mesiodistal diameters of lower permanent incisors) [mm], (95%, 75%, and 50%) (Table **2**).

### Comparative Probability Tables:

Comparison of values estimated by Moyers and by UNC Department for male patients. Space value (upper permanent canines and premolars) according to the sum of the mesiodistal diamaters of lower permanent incisors) [mm]. (95%, 75%,and 50%) (Table **3**).

### Comparative Probability Tables:

Comparison of values estimated by Moyers and by UNC Department for male patients. Space value (lower permanent canines and premolars) according to sum of the mesiodistal diameters of lower permanent incisors) [mm]. (95%, 75% and 50%) (Table **4**).

### Comparative Probability Table:

According to facial biotype. Values estimated by UNC Department. Space value (upper permanent canines and premolars) according to the sum of the mesiodistal diameters of lower permanent incisors [mm] (95%, 75%, and 50%) (Table **5**).

### Comparative Probability Table:

According to facial biotype. Values estimated by UNC Department. Space value (lower permanent canines and premolars) according to the sum of the mesiodistal diameters of lower permanent incisors [mm] (95%, 75%, and 50%) (Table **6**).

## DISCUSSION

In 1976, Moyers published his book “Orthodontic Manual for the Student and General Dentist.” In the bibliography that corresponds with chapter XI “Analysis of Dentition and Occlusion”, the author explains that his original idea of the creation of the prediction tables of the space needed for permanent canines and premolars had not been published. Therefore, the sample used for that investigation cannot be quantified and there is no evidence of the procedures used for obtaining the percentiles of his tables.

Different researchers have evaluated Moyers’ prediction tables. when applied to different ethnic groups, these tables may either underestimate or overestimate the value of the mesiodistal width of non-erupted permanent canines and premolars This demonstrates that the accuracy of these tables is debatable when applied to other ethnic groups [13, 16-26]. These results match our findings. However, Cabello Molotla CN showed that Moyers’ tables at level 75% were clinically useful when applied to the Mexican population [27].

Tathere HN *et al*. [20] suggest that Moyers’ method can be used at the 65% probability level for male subjects, and at the 75% and 85% level for upper arch and the 50% and 65% level for lower arch in female subjects. Flores- Mir *et al.* [16] observed variation in dental arch width between genders: for females, the Moyers 95^th^ percentiles in the upper arch and the 65^th^ percentiles in the lower arch predicted the sum precisely. For males, the Moyers 65^th^ percentiles in the lower arch predicted the sum with precision, but none of the Moyers percentiles provided a precise prediction in the upper arch. Melgaco *et al.* [18] stated that the predicted widths determined by Moyers´ tables at 50^th^ and 75^th^ percentiles underestimate the actual widths of the lower permanent canines and premolars for male and female patients. Abu Alhaija [19] found that there were no statistically significant differences between actual mesiodistal widths of canines and premolars and the predicted width from Moyers charts at the 65% and 75% level for the lower and upper arches in male subjects and at the 85% level for de upper and lower arches in female subjects. In our research, we have observed that Moyers’ method can be used at 95% probability level in the lower arch for female and males’ subjects

So far, no scientific studies have been conducted to show the correlation between the measured space required for permanent canines, premolars and facial biotypes, which is the reason why this research was conducted.

## CONCLUSION

A Probability Table should be created to meet the needs of the population under study without considering the patient’s facial biotype. This research clearly shows that no statistically significant differences were observed when the extent of needed space was assessed in relation to different biotypes.

Moyer´s specific probability tables (for males and females) show significant differences with those obtained in this research. Therefore, specific prediction tables should be created for men and women in each study population.

## Figures and Tables

**Table 1:** Comparative values between studies (Moyers-UNC). The differences are shown associated to a color scale, from green to red according to the magnitude of the difference. Maxillary- Female.

**Accumulated probabilities**		**Sum of the mesiodistal diameters lower central and lateral incisors**												
		19.5	20.0	20.5	21.0	21.5	22.0	22.5	23.0	**23.5**	24.0	24.5	25.0	25.5
**95%**	Moyers	21.4	21.6	21.7	21.8	21.9	22.0	22.2	22.3	22.5	22.6	22.8	22.9	23,1
	**UNC**	21.5	21.7	22.0	22.3	22.6	22.9	23.2	23.5	23.8	24.1	24.4	24.7	25,0
**75%**	Moyers	20.4	20.5	20.6	20.8	20.9	21.0	21.2	21.3	21.5	21.6	21.8	21.9	22,1
	**UNC**	20.6	20.9	21.2	21.5	21.8	22.1	22.3	22.6	22.9	23.2	23.5	23.8	24,1
**50%**	Moyers	19.6	19.8	19.9	20.1	20.2	20.3	20.5	20.6	20.8	20.9	21.0	21.2	21,3
	**UNC**	20.0	20.3	20.6	20.9	21.2	21.5	21.7	22.0	22.3	22.6	22.9	23.2	23,5

**Table of Differences**		**Sum of the mesiodistal diameters lower central and lateral incisors**												
		19.5	20.0	20.5	21.0	21.5	22.0	22.5	23.0	23.5	24.0	24.5	25.0	25.5
**95%**		-0.05	-0.15	-0.34	-0.54	-0.73	-0.92	-1.02	-1.21	-1.31	-1.50	-1.60	-1.79	-1.89
**75%**		-0.18	-0.38	-0.57	-0.67	-0.86	-1.06	-1.15	-1.34	-1.44	-1.63	-1.73	-1.92	-2.02
**50%**		-0.38	-0.47	-0.67	-0.76	-0.96	-1.15	-1.25	-1.44	-1.53	-1.73	-1.92	-2.02	-2.21

**Table 2:** Comparative values between studies (Moyers-UNC). The differences are shown associated to a color scale, from green to red according to the magnitude of the difference. Mandible - Female.

**Accumulated probabilities**		**Sum of the mesiodistal diameters lower central and lateral incisors**												
		19.5	20.0	20.5	21.0	21.5	22.0	22.5	23.0	23.5	24.0	24.5	25.0	25.5
**95%**	Moyers	20.8	21.0	21.2	21.5	21.7	22.0	22.2	22.5	22.7	23.0	23.3	23.6	23.9
	**UNC**	20.7	21.0	21.3	21.6	21.9	22.2	22.5	22.8	23.1	23.4	23.7	24.0	24.4
**75%**	Moyers	19.6	19.8	20.1	20.3	20.6	20.8	21.1	21.3	21.6	21.9	22.1	22.4	22.7
	**UNC**	19.8	20.1	20.4	20.7	21.0	21.3	21.7	22.0	22.3	22.6	22.9	23.2	23.5
**50%**	Moyers	18.7	19.0	19.2	19.5	19.8	20.0	20.3	20.5	20.8	21.1	21.3	21.6	21.8
	**UNC**	19.2	19.5	19.8	20.1	20.4	20.7	21.1	21.4	21.7	22.0	22.3	22.6	22.9

**Table of Differences**		**Sum of the mesiodistal diameters lower central and lateral incisors**												
		19.5	20.0	20.5	21.0	21.5	22.0	22.5	23.0	23.5	24.0	24.5	25.0	25.5
**95%**		0.12	0.01	-0.09	-0.10	-0.21	-0.21	-0.32	-0.32	-0.43	-0.44	-0.44	-0.45	-0.45
**75%**		-0.22	-0.32	-0.33	-0.43	-0.44	-0.55	-0.55	-0.66	-0.66	-0.67	-0.78	-0.78	-0.79
**50%**		-0.52	-0.52	-0.63	-0.63	-0.64	-0.75	-0.75	-0.86	-0.86	-0.87	-0.97	-0.98	-1.09

**Table 3:** Comparative values between studies (Moyers-UNC). The differences are shown associated to a color scale, from green to red according to the magnitude of the difference. Maxillary - Male.

**Accumulated probabilities**		**Sum of the mesiodistal diameters lower central and lateral incisors**												
		19.5	20.0	20.5	21.0	21.5	22.0	22.5	23.0	23.5	24.0	24.5	25.0	25.5
**95%**	Moyers	21.2	21.4	21.6	21.9	22.1	22.3	22.6	22.8	23.1	23.4	23.6	23.9	24.1
	**UNC**	21.8	22.1	22.4	22.6	22.9	23.2	23.4	23.7	24.0	24.3	24.5	24.8	25.1
**75%**	Moyers	20.3	20.5	20.8	21.0	21.3	21.5	21.8	22.0	22.3	22.5	22.8	23.0	23.3
	**UNC**	21.0	21.2	21.5	21.8	22.1	22.3	22.6	22.9	23.1	23.4	23.7	24.0	24.2
**50%**	Moyers	19.7	19.9	20.2	20.4	20.7	20.9	21.2	21.5	21.7	22.0	22.2	22.5	22.7
	**UNC**	20.4	20.7	20.9	21.2	21.5	21.7	22.0	22.3	22.6	22.8	23.1	23.4	23.6

**Table of Differences**		**Sum of the mesiodistal diameters lower central and lateral incisors**												
		19.5	20.0	20.5	21.0	21.5	22.0	22.5	23.0	**23.5**	24.0	24.5	25.0	25.5
**95%**		-0.62	-0.69	-0.76	-0.73	-0.80	-0.87	-0.85	-0.92	-0.89	-0.86	-0.93	-0.90	-0.97
**75%**		-0.67	-0.75	-0.72	-0.79	-0.76	-0.83	-0.80	-0.87	-0.84	-0.91	-0.88	-0.96	-0.93
**50%**		-0.69	-0.76	-0.73	-0.80	-0.77	-0.84	-0.81	-0.78	-0.86	-0.83	-0.90	-0.87	-0.94

**Table 4:** Comparative values between studies (Moyers-UNC). The differences are shown associated to a color scale, from green to red according to the magnitude of the difference. Mandible - Male.

**Accumulated probabilities**		**Sum of the mesiodistal diameters lower central and lateral incisors**												
		19.5	20.0	20.5	21.0	21.5	22.0	22.5	23.0	23.5	24.0	24.5	25.0	25.5
**95%**	Moyers	21.6	21.8	22.0	22.2	22.4	22.6	22.8	23.0	23.2	23.5	23.7	23.9	24.2
	**UNC**	21.2	21.5	21.7	22.0	22.3	22.6	22.8	23.1	23.4	23.6	23.9	24.2	24.5
**75%**	Moyers	20.4	20.6	20.8	21.0	21.2	21.4	21.6	21.9	22.1	22.3	22.5	22.8	23.0
	**UNC**	20.4	20.7	21.0	21.2	21.5	21.8	22.1	22.3	22.6	22.9	23.1	23.4	23.7
**50%**	Moyers	19.5	19.7	20.0	20.2	20.4	20.6	20.9	21.1	21.3	21.5	21.7	22.0	22.2
	**UNC**	19.9	20.2	20.4	20.7	21.0	21.2	21.5	21.8	22.1	22.3	22.6	22.9	23.2

**Table of Differences**		**Sum of the mesiodistal diameters lower central and lateral incisors**												
		19.5	20.0	20.5	21.0	21.5	22.0	22.5	23.0	**23.5**	24.0	24.5	25.0	25.5
**95%**		0.41	0.34	0.27	0.19	0.12	0.05	-0.03	-0.10	-0.17	-0.15	-0.22	-0.30	-0.27
**75%**		-0.01	-0.09	-0.16	-0.23	-0.31	-0.38	-0.46	-0.43	-0.50	-0.58	-0.65	-0.62	-0.70
**50%**		-0.38	-0.45	-0.42	-0.50	-0.57	-0.65	-0.62	-0.69	-0.77	-0.84	-0.91	-0.89	-0.96

**Table 5:** Probability table according facial to biotype (*P* 95%; 75%; 50%).

***P***	**Biotype**	**Sum of the mesiodistal diameters lower central and lateral incisors**																						
		**18,0**	**18,5**	**19,0**	**19,5**	**20,0**	**20,5**	**21,0**	**21,5**	**22,0**	**22,5**	**23,0**	**23,5**	**24,0**	**24,5**	**25,0**	**25,5**	**26,0**	**26,5**	**27,0**	**27,5**	**28,0**	**28,5**	**29,0**
																								
**95%**	DOLICHOFOCIAL	20,1	20,5	20,8	21,1	21,5	21,8	22,1	22,5	22,8	23,1	23,4	23,8	24,1	24,4	24,8	25,1	25,4	25,8	26,1	26,4	26,8	27,1	27,4
	MESOFACIAL	20,6	20,9	21,2	21,5	21,8	22,1	22,4	22,7	23,0	23,2	23,5	23,8	24,1	24,4	24,7	25,0	25,3	25,6	25,9	26,2	26,4	26,7	27,0
	BRAQUIFACIAl	20,7	20,9	21,2	21,5	21,8	22,1	22,3	22,6	22,9	23,2	23,4	23,7	24,0	24,3	24,6	24,8	25,1	25,4	25,7	26,0	26,2	26,5	26,8
																								
**75%**	DOLICHOFOCIAL	19,4	19,7	20,0	20,4	20,7	21,0	21,4	21,7	22,0	22,4	22,7	23,0	23,3	23,7	24,0	24,3	24,7	25,0	25,3	25,7	26,0	26,3	26,7
	MESOFACIAL	19,8	20,1	20,4	20,7	20,9	21,2	21,5	21,8	22,1	22,4	22,7	23,0	23,3	23,6	23,9	24,1	24,4	24,7	25,0	25,3	25,6	25,9	26,2
	BRAQUIFACIAl	19,9	20,2	20,4	20,7	21,0	21,3	21,5	21,8	22,1	22,4	22,7	22,9	23,2	23,5	23,8	24,0	24,3	24,6	24,9	25,2	25,4	25,7	26,0
																								
**50%**	DOLICHOFOCIAL	18,9	19,2	19,5	19,8	20,2	20,5	20,8	21,2	21,5	21,8	22,2	22,5	22,8	23,1	23,5	23,8	24,1	24,5	24,8	25,1	25,5	25,8	26,1
	MESOFACIAL	19,2	19,5	19,8	20,1	20,4	20,6	20,9	21,2	21,5	21,8	22,1	22,4	22,7	23,0	23,3	23,6	23,8	24,1	24,4	24,7	25,0	25,3	25,6
	BRAQUIFACIAl	19,3	19,6	19,9	20,2	20,4	20,7	21,0	21,3	21,6	21,8	22,1	22,4	22,7	22,9	23,2	23,5	23,8	24,1	24,3	24,6	24,9	25,2	25,5

**Table 6:** Probability table according to facial biotype (*P* 95%; 75%; 50%).

***P***	**Biotype**	**Sum of the mesiodistal diameters lower central and lateral incisors**																						
		**18,0**	**18,5**	**19,0**	**19,5**	**20,0**	**20,5**	**21,0**	**21,5**	**22,0**	**22,5**	**23,0**	**23,5**	**24,0**	**24,5**	**25,0**	**25,5**	**26,0**	**26,5**	**27,0**	**27,5**	**28,0**	**28,5**	**29,0**
																								
**95%**	DOLICHOFOCIAL	19,1	19,5	19,8	20,2	20,6	20,9	21,3	21,7	22,0	22,4	22,8	23,1	23,5	23,9	24,2	24,6	25,0	25,3	25,7	26,1	26,4	26,8	27,2
	MESOFACIAL	20,1	20,4	20,7	21,0	21,2	21,5	21,8	22,1	22,4	22,7	23,0	23,3	23,6	23,9	24,2	24,5	24,8	25,1	25,4	25,7	26,0	26,3	26,6
	BRAQUIFACIAl	20,0	20,3	20,5	20,8	21,1	21,4	21,7	22,0	22,3	22,6	22,9	23,1	23,4	23,7	24,0	24,3	24,6	24,9	25,2	25,5	25,7	26,0	26,3
																								
**75%**	DOLICHOFOCIAL	18,4	18,7	19,1	19,5	19,8	20,2	20,6	20,9	21,3	21,7	22,0	22,4	22,8	23,1	23,5	23,9	24,2	24,6	25,0	25,3	25,7	26,1	26,4
	MESOFACIAL	19,2	19,5	19,8	20,1	20,4	20,7	21,0	21,3	21,6	21,9	22,1	22,4	22,7	23,0	23,3	23,6	23,9	24,2	24,5	24,8	25,1	25,4	25,7
	BRAQUIFACIAl	19,2	19,5	19,8	20,1	20,4	20,6	20,9	21,2	21,5	21,8	22,1	22,4	22,7	23,0	23,2	23,5	23,8	24,1	24,4	24,7	25,0	25,3	25,6
																								
**50%**	DOLICHOFOCIAL	17,9	18,3	18,6	19,0	19,4	19,7	20,1	20,5	20,8	21,2	21,6	21,9	22,3	22,6	23,0	23,4	23,7	24,1	24,5	24,8	25,2	25,6	25,9
	MESOFACIAL	18,6	18,9	19,2	19,5	19,8	20,1	20,4	20,7	20,9	21,2	21,5	21,8	22,1	22,4	22,7	23,0	23,3	23,6	23,9	24,2	24,5	24,8	25,1
	BRAQUIFACIAl	18,7	19,0	19,3	19,5	19,8	20,1	20,4	20,7	21,0	21,3	21,6	21,9	22,1	22,4	22,7	23,0	23,3	23,6	23,9	24,2	24,5	24,7	25,0
